# Supplementary material for: Major advance of South Georgia glaciers during the Antarctic Cold Reversal following extensive sub-Antarctic glaciation
Source: Nat Commun. 2017 Mar 17;8:14798. doi: 10.1038/ncomms14798 (PMC5357866; doi:10.1038/ncomms14798)
Supplement: Supplementary Information — Supplementary Figures and Supplementary References [file ncomms14798-s1.pdf]

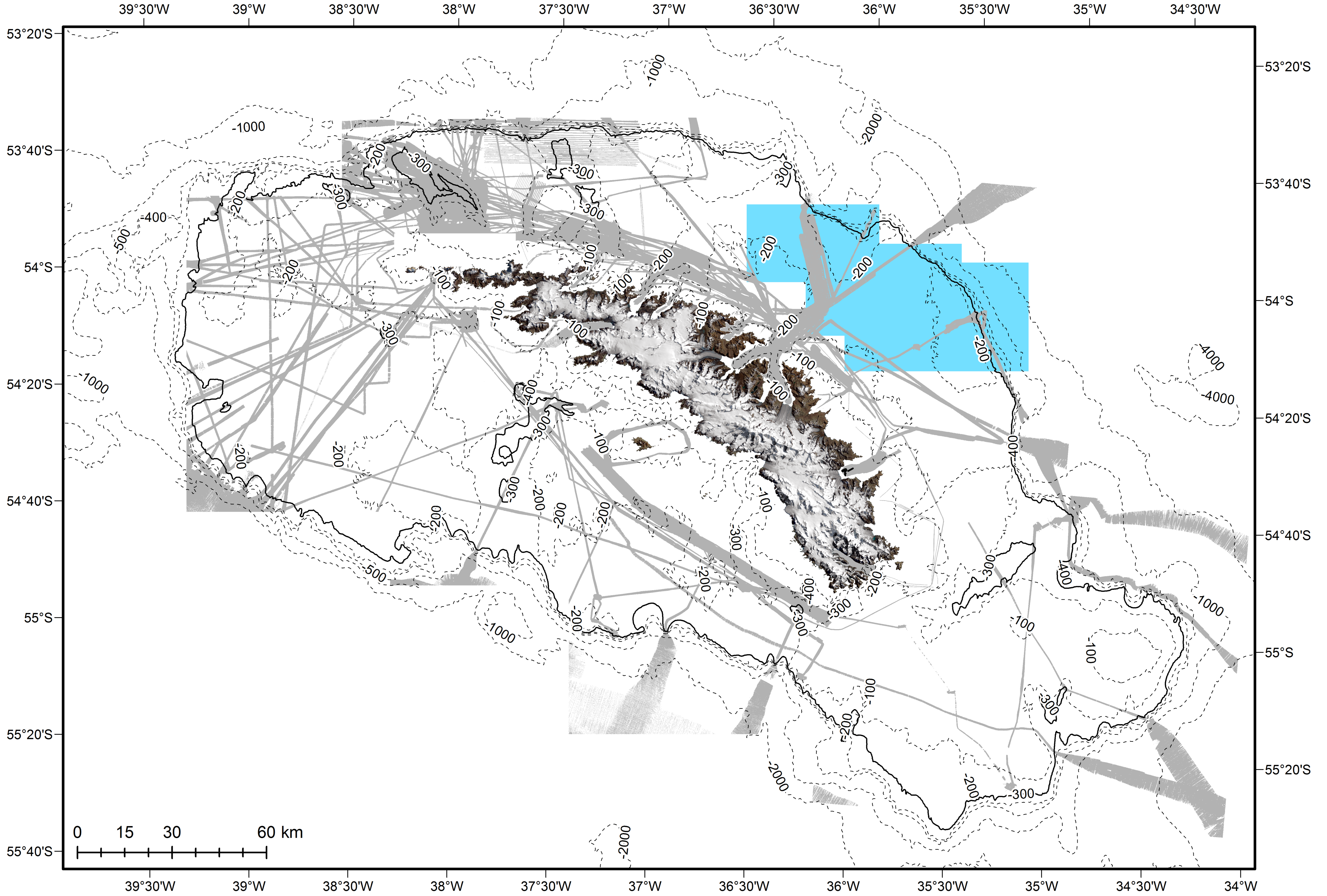

Supplementary figure 1: Coverage of gridded multibeam datasets used for mapping on the South Georgia continental block (solid grey). Tiles of Olex singlebeam gridded data are also shown (light blue) though data coverage within these sub-areas is only partial. Contours derived from regional bathymetry dataset in ref 1.

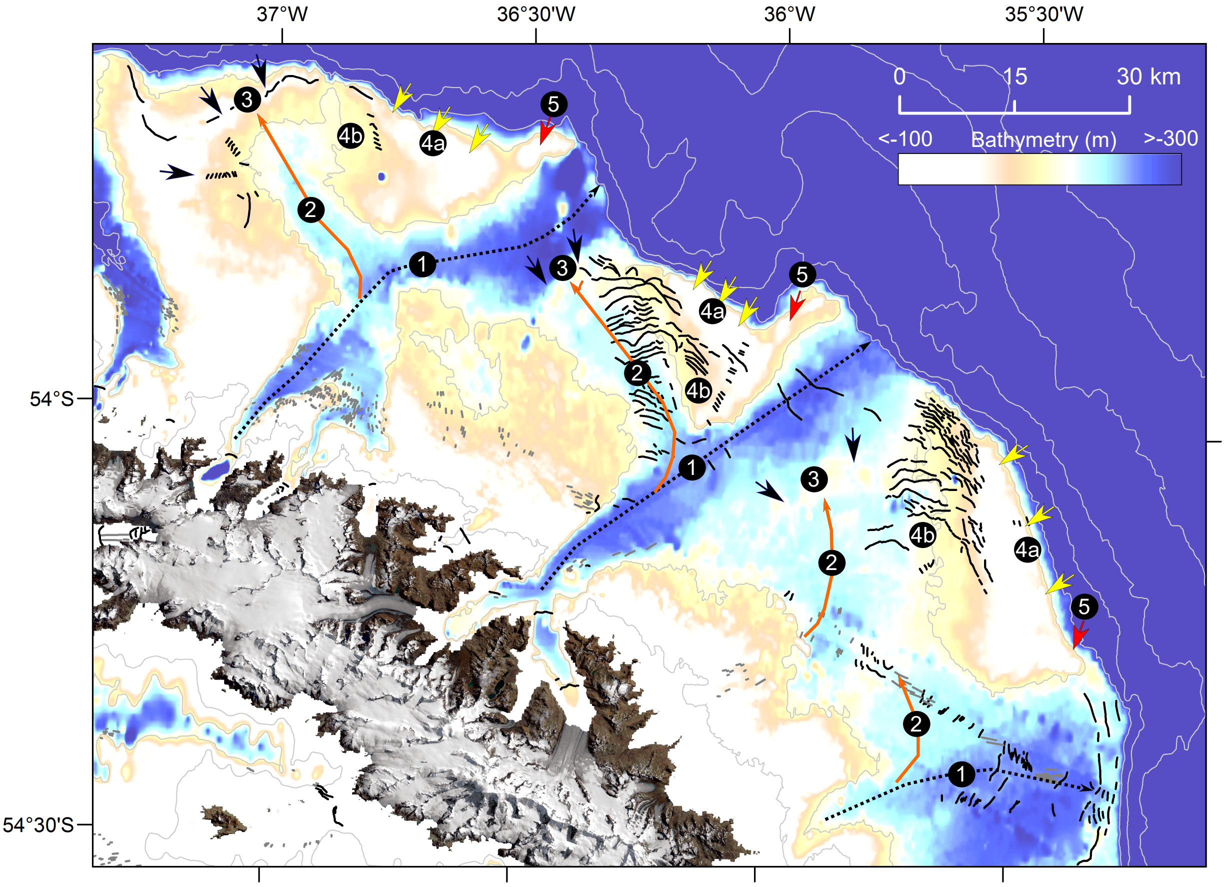

Supplementary Figure 2: Bathymetry of the northern South Georgia continental shelf showing broad-scale geomorphic elements indicative of shared, complex ice-sheet history. Main linear trough systems are identified (1; dashed black lines), with secondary shallower troughs veering to the west (2; orange arrows). These secondary troughs terminate at lobate or crescent-shaped ridges, interpreted as moraines (3; black arrows). Larger shelf-edge banks on the western flank of the main troughs (4a; yellow arrows) which are, in turn, overprinted by sequences of smaller ridges (4b; black lines). The shelf edge geometry at the western terminus of each trough shows a clear 'step' or promontory (5; red arrows). The consistent large-scale geometries suggest a common history of evolution and formation through multiple glacial cycles.

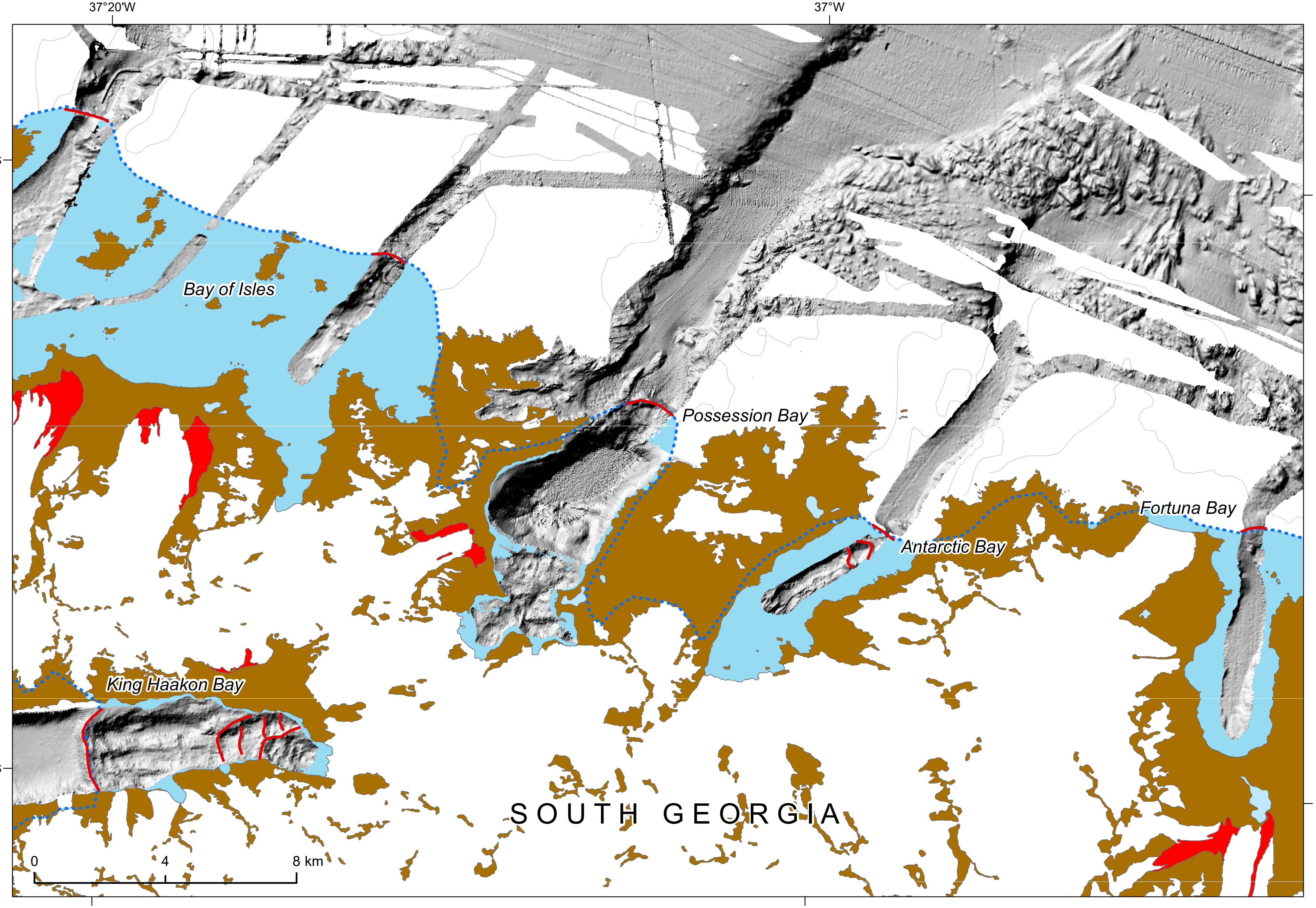

Supplementary Figure 3 : Bathymetry of the north-western South Georgia fjords highlighting a common fjord-mouth moraine, which is interpreted as the expression of ACR advance around the island. Moraines shown in red; modern-day ice cover in white. Interpreted ACR ice-cap extent shown in blue. Bathymetry shown as a grey-scale hillshade only, to accentuate submarine geomorphology. ACR: Antarctic Cold Reversal.

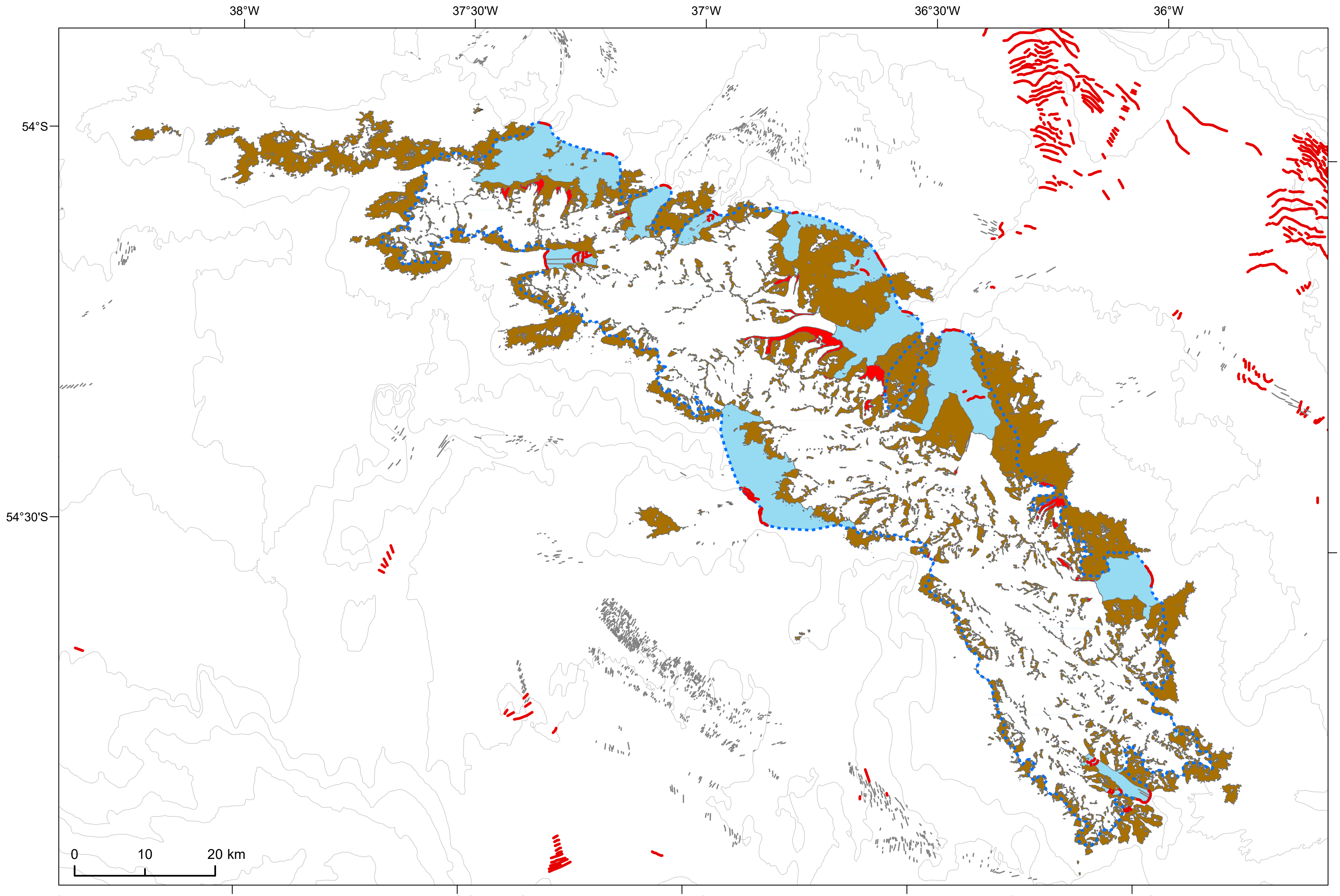

Supplementary Figure 4: Tentative reconstruction of South Georgia ice cap extent (blue shaded with bold blue dashed line) at the Antarctic Cold Reversal, based on fjord-mouth geomorphic features. Where features do not constrain the limit, ice margins have been drawn at or close to present-day ice positions. Thus, the area of reconstructed glacial ice cover is conservative. Moraines shown in red; modern-day ice cover in white; streamlined landforms at the sea bed shown as short grey lines.

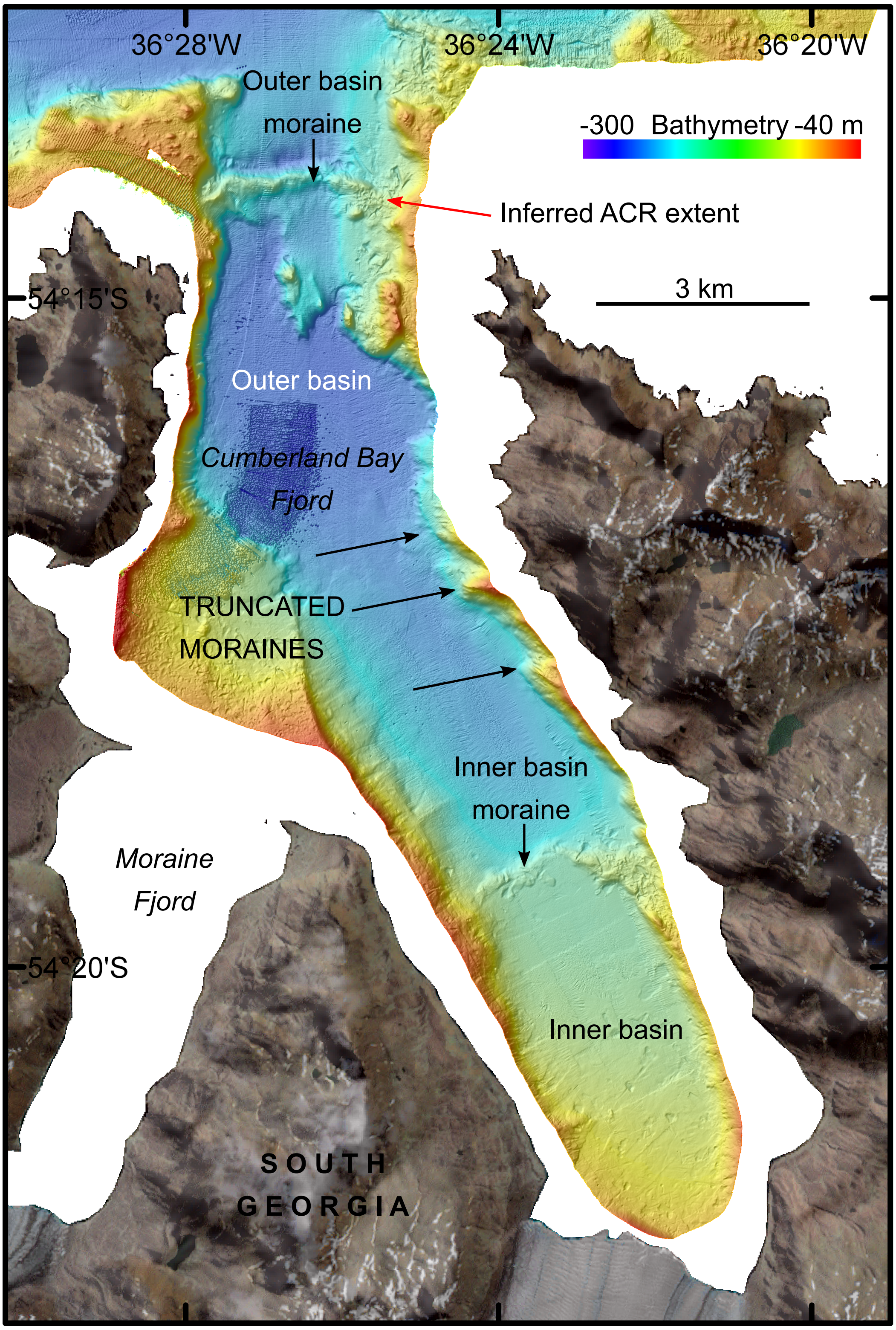

Supplementary Figure 5: Multibeam bathymetry of Cumberland Bay fjord, showing eroded promontories flanking the outer fjord basin, interpreted as truncated moraines.

Supplementary References

1. Fretwell, P. T. *et al.* Compilation of a new bathymetric dataset of South Georgia. *Antarct Sci* **21**, 171-74, (2009).
